# The music of the genes

**DOI:** 10.1186/2041-2223-5-2

**Published:** 2014-01-22

**Authors:** Mark A Jobling

**Affiliations:** 1 Department of Genetics, University of Leicester, University Road, Leicester LE1 7RH, UK

## 

The most famous of musical dynasties is that of the Bachs. In Leipzig for a conference, I visited the Bach Museum opposite the Thomaskirche where the greatest Bach of all worked as Kapellmeister for 27 years, and is now buried. There, the family’s many names are laid out on a wall, from Johann Sebastian’s great-great-grandfather Veit, a miller and player of the cittern, born around 1550, via J.S. himself, and on to his grandson Wilhelm Friedrich Ernst. One by one, the names light up, and as each Bach is illuminated his music fills the air.

The occurrence of so many great musicians in seven generations of one family might suggest musical ability in the genes. Francis Galton, in his *Hereditary Genius*[1], studied musicians alongside judges, statesmen, scientists, commanders, authors, poets and artists, as one of his classes of ‘eminent men’ (note, no women). As well as these brainy and artistic types, he cast wrestlers and rowers into the mix, too. Galton investigated the pattern of inheritance of exceptional ability within families, showing that the eminence of relatives of an eminent man declined with the degree of relatedness, and taking this as evidence of heritability.

There are two difficulties with Galton’s approach to musical ability - the phenotype (general eminence) is complex and vaguely defined, and the influence of the environment is not accounted for. In the Bach family, for example, musical training at a young age was the norm, and clearly led to a good living, so alternative careers were not necessarily high on the agenda.

Modern geneticists have also been interested in questions of musical inheritance, and have mostly focused on a simpler phenotype, absolute pitch (AP) - the ability to instantly recognize and correctly name the pitch of any of about seventy different notes in the middle of the auditory range. The phenotype is generally rare, and found in only approximately 1% to 2% of music students and musical professionals, so is far from being a proxy for musicianship. Clearly, AP requires some prior exposure to notes and their names, and an ability to fix and recall these associations. Oliver Sacks, in a chapter of his book *Musicophilia*[2] entitled ‘Papa Blows his Nose in G’, points out that the real wonder of AP is that for people who possess it, each tone has its own unique characteristic (for example, F-sharp-ness), which to them is often analogous to colour. Indeed, some composers, including Scriabin and Messiaen, explicitly linked notes and colours, possible examples of synesthesia, in which one kind of sensory stimulus evokes another.

AP is a complex trait, involving both genetic and environmental factors: musical training during early development contributes to its acquisition, and it is more frequent in Asians than Europeans, a finding that some have attributed to early exposure to languages in which tone is particularly important. Genome-wide linkage analysis [3] in families showing both AP and synesthesia highlighted a shared region on chromosome 6, supporting the relationship between these phenotypes. Subsequent sequencing of candidate genes revealed otherwise rare amino-acid-changing variants in affected members of four families in *EPHA7*, a gene encoding a member of a family of cell-surface-bound receptor tyrosine kinases that may play an important role in neural differentiation and connectivity in the developing brain.

One particular genetically defined group appears to exhibit a high natural ability for music. These are people with Williams-Beuren syndrome (WBS [4]). Carrying a 1.5- to 1.8-Mb deletion on the long arm of chromosome 7 that removes 26 to 28 genes, subjects suffer from a constellation of abnormalities, with a mean IQ of 55, and severe difficulties in spatial tasks such as solving jigsaw puzzles. However, there are compensating strengths in musical ability, with many showing skill in singing or playing instruments. There is disagreement about the incidence of absolute pitch in WBS: in a study by neuroscientist Howard Lenhoff [5], five young WBS subjects all had AP, but a more recent larger study has failed to find a convincing association [6]. Lenhoff’s own daughter, Gloria, has WBS and is a musical savant, singing almost 2,000 songs in many languages from memory, and performing with renowned orchestras.

As well as phenotypes that enhance musical abilities, there are some that do the opposite. Congenital amusia (or tone deafness) is the failure to acquire the perception and recognition of music, despite having normal hearing, language, and intelligence. One patient seen by Sacks [2] could not recognize ‘Happy Birthday to You’, even though, as a school-teacher, she was obliged to play a recording of it at least 30 times a year. To her, the sound of music was ‘like pots and pans being thrown on the floor’. Family studies [7] show a genetic component, since 39% of first-degree relatives of subjects have amusia, compared to a population frequency of about 4%, but no gene hunts have yet been undertaken.

Setting aside these unfortunate tone-deaf cases, music is a cultural universal, and findings of prehistoric bone flutes in southern Germany show that humans have been making sophisticated music for at least 42,000 years [8,9]. But what is it all for? Darwin believed that music evolved through sexual selection; by analogy with birds and their songs, the creation and appreciation of music was part of the complex process of attracting the opposite sex. ‘Music has a wonderful power … of recalling in a vague and indefinite manner, those strong emotions which were felt during long-past ages, when, as is probable, our early progenitors courted each other by the aid of vocal tones’ [10]. In Darwin’s opinion, language came after music. Herbert Spencer disagreed, claiming that music arose naturally from the cadences used in emotional speech. In modern times, Steven Pinker [11] has sided with Spencer, arguing that music is simply a useless byproduct of language (‘auditory cheesecake’). Steven Mithen [12], however, believes that music is too different from language to be a byproduct, and that its emotional power indicates a long and important evolutionary history. A consensus seems unlikely to emerge any time soon.

The Leipzig conference in which I participated was concerned with language, and I learned that linguists disagree violently about some fundamental aspects of what modern languages can tell us about populations in the distant past. Funnily enough, music might help here. Analysing characters such as rhythm, pitch and dynamics in traditional vocal songs from nine indigenous populations of Thailand [13] allows a music-based distance measure to be calculated between them. Comparison of this to analogous distances based on languages and genetics (using maternally inherited mitochondrial DNA) shows that music is a better fit to genetics than language is. Likewise, a geographically broader study of Eurasian populations [14] shows that high musical similarity predicts high genetic similarity, and that the relationship is stronger for maternal than for paternal lineages.

Perhaps, then, musicology could replace historical linguistics as a tool to find cultural connections that reflect deep shared ancestry - the language changes, but (like the legacy of Bach) the melody lingers on. Mothers sing to their babies, after all.

## References

[B1] Galton F Hereditary Genius: An Enquiry into its Laws and Consequences 1869 London: Macmillan

[B2] Sacks O Musicophilia: Tales of Music and the Brain 2007 London: Picador

[B3] Gregersen PK Kowalsky E Lee A Baron-Cohen S Fisher SE Asher JE Ballard D Freudenberg J Li W Absolute pitch exhibits phenotypic and genetic overlap with synesthesia Hum Mol Genet 2013 22 2097 2104 10.1093/hmg/ddt059 23406871 PMC4707203

[B4] Pober BR Williams-Beuren syndrome N Engl J Med 2010 362 239 252 10.1056/NEJMra0903074 20089974

[B5] Lenhoff HM Perales O Hickok G Absolute pitch in Williams syndrome Music Percep 2001 18 491 503 10.1525/mp.2001.18.4.491

[B6] Martinez-Castilla P Sotillo M Campos R Do individuals with Williams syndrome possess absolute pitch? Child Neuropsychol 2013 19 78 96 10.1080/09297049.2011.639755 22145764

[B7] Peretz I Cummings S Dube MP The genetics of congenital amusia (tone deafness): a family-aggregation study Am J Hum Genet 2007 81 582 588 10.1086/521337 17701903 PMC1950825

[B8] Conard NJ Malina M Munzel SC New flutes document the earliest musical tradition in southwestern Germany Nature 2009 460 737 740 19553935 10.1038/nature08169

[B9] Higham T Basell L Jacobi R Wood R Ramsey CB Conard NJ Testing models for the beginnings of the Aurignacian and the advent of figurative art and music: the radiocarbon chronology of Geissenklosterle J Hum Evol 2012 62 664 676 10.1016/j.jhevol.2012.03.003 22575323

[B10] Darwin C The Expression of the Emotions in Man and Animals 1872 London: John Murray

[B11] Pinker S How the Mind Works 1997 London: Penguin

[B12] Mithen S The Singing Neanderthals: The Origins of Music, Language, Mind and Body 2005 London: Weidenfeld & Nicolson

[B13] Brown S Savage PE Ko AM Stoneking M Ko YC Loo JH Trejaut JA Correlations in the population structure of music, genes and language Proc R Soc B 2014 281 20132072 24225453 10.1098/rspb.2013.2072 PMC3843827

[B14] Pamjav H Juhasz Z Zalan A Nemeth E Damdin B A comparative phylogenetic study of genetics and folk music Mol Genet Genomics 2012 287 337 349 10.1007/s00438-012-0683-y 22392540

